# Progress in Otology

**Published:** 1903-04-25

**Authors:** 


### PROGRESS IN OTOLOGY.

(*Continued from page* 45.)

**Ozone in Chronic Middle-ear Deafness.**—This is the title of a short paper by George Stoker.[4] It is to the form of deafness popularly known as "throat-deafness," or to the profession as "chronic dry catarrh of the middle ear," generally believed to be due to stenosis of the Eustachian tube, that the paper refers. The effect of oxygen and ozone in restoring a healthy condition to the nasal mucous membrane led to a trial of the remedy for this form of chronic progressive deafness. The ozone was generated by means of Rhumkorff's coil to which the ozonising tube was attached. The ozone was pumped into the middle ear through a Eustachian catheter for about three minutes, from twice to four times a week. In all the four cases mentioned the hearing was considerably improved and the tinnitus disappeared after a few applications.

**Suppuration of the Middle Ear.**—This subject was recently discussed in the Medical Society of New York.[5] J. B. Clemens remarked that mastoid periostitis was frequently observed as a result of middle-ear suppuration. Inflammation of the mastoid cells was apt to occur as a complication when there was delayed perforation of the drum and deficient drainage. Retropharyngeal abscess might occur through an extrusion of pus through the Eustachian tube. A more or less localised meningitis was almost always associated with acute suppurative otitis media in young children. W. C. Phillips said that rest in bed acted as a powerful abortive measure in acute cases. Free purgation with calomel was useful and the pain could to some extent be relieved and the inflammation checked by irrigation of the ear at intervals of half an hour with sterile water at a temperature of 100° to 110°F. If the drum membrane bulged it should be properly incised rather than punctured. The ice-coil should not be used for more than twenty-four or thirty-six hours. Early operation was usually followed by complete restoration of hearing. Under appropriate treatment the cases always recovered in from two days to three weeks. J. F. McKernon discussed chronic suppuration, defining it as that variety which had existed for six months or more. Usually the higher the perforation in the drum the greater the involvement of the structures beyond. The "wet" method of treatment was the safer. For irrigation the following solutions might be used, viz., bichloride of mercury 1 in 4,000 to 1 in 8,000, boric acid, 20 grains to the ounce, carbolic acid of a one or two per cent. strength, or a weak solution of formaldehyde or of permanganate of potash. By the Valsalva method the discharge could be dislodged from the less accessible parts. Of the astringent solutions used, that of nitrate of silver, 5 grains to the ounce, was the most popular.

**Purulent Otitis in Infants.**—Max Toeplitz[6] has discussed this subject. He points out that in infants the tympanic ring is much shorter than in the adult, the antrum and middle ear are nearer the surface of the skull than later in life, and the Eustachian tube is shorter and more horizontal. In some cases of the affection under consideration the very high temperature and the presence of stupor or of convulsions suggested cerebral disease. A positive diagnosis of meningitis could only be made by lumbar puncture. The acute stage lasted from a few hours to a week or more, and terminated in spontaneous perforation or in chronic otitis or in death from meningitis. In children primary inflammation of the labyrinth manifested itself by fever, convulsions, a tendency to vomiting, dulness of the sensorium, stiffness of the neck, and an irregular pulse. In children the petrous bones were rapidly destroyed. The treatment advocated was on the lines of that described above for acute otitis in adults, but the dry method was preferred if it could be properly supervised.

**The Indications for the Mastoid Operation.**—Macleod Yearsley[7] has contributed a paper on this subject. Operations on the mastoid, at first merely perforations, appear to have been performed for rather more than a century. The "complete post-aural mastoid operation," if called by any other name should be styled the "combined Schwartze-Stäcke" operation. The operations as now performed belong to one of two groups: (1) the opening of the mastoid antrum without interfering with the middle ear, and (2) the opening of the antrum and the formation of one large cavity common to it and the tympanum. Speaking roughly the former is adapted for the relief of acute cases and the latter for the cure of chronic suppuration. The signs of acute mastoid disease are not always very distinct. As an indication of the complication discharge cannot be relied upon unless it is very profuse and unless after syringing pus can again be seen pouring from the region of the attic. The formation of an abscess over the mastoid should not be waited for. Pus may pass to the surface from the tympanum behind the cartilaginous meatus without involving the mastoid at all. In the latter case the swelling is characteristic in that it always obliterates the retro-auricular groove, whereas in true mastoid involvement this groove is preserved although the pinna may be displaced. Bulging of the superior-posterior wall of the meatus at its inner end is pathognomonic of antrum disease. Tenderness at the tip of the mastoid indicates rather periostitis than antral abscess. Three rare symptoms, if present, are highly suggestive of mastoid disease, viz, pain on mastication, lateral nystagmus due to osteitis about the horizontal semi-circular canal, and rigor. Schwartze's operation of simply opening and draining the antrum is the operation usually required for acute mastoid suppuration. If the acute mastoiditis is the result of an exacerbation in the course of a chronic middle-ear suppuration, the complete post-aural operation is indicated. Influenzal mastoiditis is one of the most destructive in type, and arises either primarily—due apparently to direct infection and not to extension—or secondarily to middle-ear suppuration. In the former case the mastoiditis is not preceded by ear discharge. Prompt treatment is required owing to the great tendency to the quick production of caries and intracranial complications. Yearsley has seen the whole mastoid destroyed by this form of inflammation in less than a fortnight. The following chronic conditions requiring a mastoid operation usually need the complete form of it:—(1) Chronic otorrhœa, (2) recurring discharge, (3) facial paralysis, (4) cholesteatoma, (5) persistent mastoid pain, (6) meatal stenosis, (7) Bezold's mastoiditis, (8) mastoid fistula, (9) necrosis.

**Tuberculous Otitis Media.**—G. K. Grimmer[8] has contributed an article on this subject. Tubercle bacilli may find their way to the tympanum (1) mechanically or by extension along the Eustachian tube, (2) by the blood or lymphatic vessels, (3) through a perforation of the tympanic membrane. The affection may be a primary or secondary one. The clinical course may be acute or chronic. Usually the onset is insidious, with little pain and only a slight discharge. Perforations are frequently multiple, and are circular with thick œdematous edges. Early destruction of bone is common, and hence facial paralysis is a common symptom. To clinch the diagnosis the bacilli should be sought for in the granulation tissue, or inoculation experiments made. The prognosis is unfavourable. Some cases must be regarded as hopeless from the first, *e.g.*, cases in infants with emaciation, facial paralysis, copious blood-stained discharge, and masses of enlarged glands round the ear. Many cases can be benefited by operation. The disease is more frequent in persons under five years of age than above. Bare bone can be felt more frequently through the meatus in tuberculous disease than in other cases, and the formation of sequestra is frequent. In the author's cases facial paralysis was twice as common in this form of otitis as in others, and in none did the discharge follow an acute illness. Enlarged glands were felt near the affected ear in all the tuberculous cases. A suppurating sinus near the ear, with pale ademalous granulations, is strong evidence that the lesion is tuberculous. The author estimates that 65 to 70 per cent. of cases of suppurative otitis, with neighbouring bone lesion in children under five, are tuberculous; in older persons not more than 16 per cent. are tuberculous. The presence of cholesteatomatous masses is opposed to tubercles being present. Milligan[9] read a paper on this subject recently at the Otological Society. He concluded that the disease was usually a secondary one, but that as a primary affection it was more common than was usually supposed. The prognosis was always grave, but in a certain proportion of cases the disease could be eradicated by surgical means. If less than 10 per cent. of hearing-power remained no attempt should be made to preserve the organ as an organ of special sense.

[4] Lancet, Nov. 1, 1902, p 1187. [5] Med. Rec, Oct. 11, 1902, p. 592. [6] Ibid, Jan. 17, 1903, p. 118. [7] Med. Times and Hosp. Gaz., Jan. 17, 1903, p. 33. [8] Vide Abst. Practitioner, November 1902, p. 613. [9] Lancet, Feb. 21, 1903, p. 522.

(*To be concluded.*)

